# Health-Related Quality of Life in Pregnancy Associated with Psychological Distress

**DOI:** 10.3390/medicina62030445

**Published:** 2026-02-26

**Authors:** Brenda-Cristiana Bernad, Mirela-Cleopatra Tomescu, Dana Emilia Velimirovici, Minodora Andor, Diana Lungeanu, Virgil Enătescu, Andreea Luciana Rata, Sergiu-Florin Arnautu, Elena Silvia Bernad, Oana Neda-Stepan, Lavinia Hogea

**Affiliations:** 1 Department of Neuroscience, Doctoral School, Centre for Neuropsychology and Behavioural Medicine, Multidisciplinary Heart Research Centre, “Victor Babeș” University of Medicine and Pharmacy from Timișoara, 300041 Timișoara, Romania; bernad.brenda@umft.ro; 2 Department of Internal Medicine, Centre for Multidisciplinary Heart Research, “Victor Babeș” University of Medicine and Pharmacy from Timișoara, 300041 Timișoara, Romaniaandor.minodora@umft.ro (M.A.); 3 Cardiology Clinics, Timișoara Clinical Municipal Emergency Hospital, 300040 Timișoara, Romania; 4 Department of Cardiology, “Victor Babes” University of Medicine and Pharmacy, 300041 Timișoara, Romania; dana.velimirovici@umft.ro; 5 Institute of Cardiovascular Diseases Timișoara, 300310 Timișoara, Romania; 6 Center for Modeling Biological Systems and Data Analysis, “Victor Babeș” University of Medicine and Pharmacy from Timișoara, 300041 Timișoara, Romania; dlungeanu@umft.ro; 7 Department of Neuroscience, “Victor Babeș” University of Medicine and Pharmacy from Timișoara, 300041 Timișoara, Romania; enatescu.virgil@umft.ro (V.E.); oana.neda-stepan@umft.ro (O.N.-S.); 8 Clinic of Psychiatry, “Pius Brînzeu” County Clinical Emergency Hospital, 300723 Timișoara, Romania; 9 Department of Surgical Emergencies, “Victor Babeș” University of Medicine and Pharmacy from Timișoara, 300041 Timișoara, Romania; andreea.rata@umft.ro; 10 Department of Vascular Surgery, “Pius Brînzeu” County Clinical Emergency Hospital, 300723 Timișoara, Romania; 11 Department of Internal Medicine, Centre for Cognitive Research in Neuropsychiatric Pathology, Centre for Multidisciplinary Heart Research, “Victor Babeș” University of Medicine and Pharmacy from Timișoara, 300041 Timișoara, Romania; arnautu.sergiu@umft.ro; 12 Department of Obstetrics and Gynecology, Center for Laparoscopy, Laparoscopic Surgery and In Vitro Fertilization, Centre for Neuropsychology and Behavioural Medicine, “Victor Babeș” University of Medicine and Pharmacy from Timișoara, 300041 Timișoara, Romania; 13 First Clinic of Obstetrics and Gynecology, Laparoscopy, In Vitro Fertilization and Embryotransfer Research Center, “Pius Brînzeu” County Clinical Emergency Hospital, 300723 Timișoara, Romania; 14 Department of Neuroscience, Centre for Neuropsychology and Behavioural Medicine, “Victor Babeș” University of Medicine and Pharmacy from Timișoara, 300041 Timișoara, Romania; hogea.lavinia@umft.ro

**Keywords:** psychological distress, pregnancy, quality of life, high risk pregnancy, alcohol consumption, tobacco consumption

## Abstract

*Background and Objectives*: Pregnancy is associated with profound physical and psychological changes in a woman’s life. Psychological distress and medical comorbidities during pregnancy remain under recognized despite their potential impact on maternal well-being. This study aimed to examine the associations between psychological distress, physical and mental components of health-related quality of life (HRQoL), lifestyle factors (alcohol and tobacco use), and the presence of medical comorbidities in pregnant women. *Materials and Methods:* A cross-sectional study was conducted among pregnant women in the second and third trimesters admitted to a tertiary obstetrics and gynecology center in Romania. Psychological distress was assessed using the Symptom Checklist-90-Revised (SCL-90-R) Global Severity Index (GSI), while health-related quality of life (HRQoL) was evaluated with the Short Form Health Survey-36 items (SF-36) physical (PCS) and mental (MCS) Component Summary scores. Alcohol and tobacco use were assessed using Alcohol Use Disorders Identification Test—Consumption (AUDIT-C) and the Fagerström Test for Nicotine Dependence (FTND). Nonparametric tests were used for correlation and group-comparison analyses. *Results*: Among the 337 valid answers, higher psychological distress was significantly associated with lower physical (R = −0.16, *p* < 0.01) and mental (R = −0.26, *p* < 0.01) HRQoL. Pregnant women with medical comorbidities reported higher psychological distress and poorer physical HRQoL compared with those without comorbidities, while mental HRQoL did not differ significantly. Alcohol and tobacco use were not significantly associated with HRQoL or psychological distress. *Conclusions*: Psychological distress is a central factor associated with both physical and mental quality of life during pregnancy. Integrating routine mental health screening into antenatal care, particularly for women with medical comorbidities, may improve maternal well-being and support better pregnancy outcomes.

## 1. Introduction

### 1.1. Background

Pregnancy represents a sensitive period in which maternal mental health extends its influence beyond the mother to the developing fetus [1]. Psychological well-being supports normal pregnancy adaptation and contributes to favorable perinatal outcomes. In contrast, early disturbances in mental health have been shown to affect fetal development from the initial stages of gestation [2,3]. More effective care pathways, including early interventions, are needed to reduce the negative impact and ensure a successful pregnancy and birth [4]. These effects underline why maternal mental health is increasingly recognized as a key component of antenatal risk assessment. Recent studies also demonstrate a link between maternal mental health during pregnancy and the likelihood of preterm birth and small for gestational age, demonstrating that poor maternal mental health is a significant risk factor for these complications [5].

Alongside mental health, physical functioning represents an essential component of maternal well-being. Physical health and psychological distress are closely related during pregnancy, and maternal physical status has been associated with pregnancy complications such as hypertensive disorders, gestational diabetes, and preterm birth [6,7,8]. Maternal physical well-being encompasses general health perception, bodily pain, fatigue, and mobility.

Psychological stress during pregnancy can also influence fetal development through hormonal and inflammatory pathways [9]. Elevated maternal cortisol and cytokine levels can cross the placenta, potentially disrupting fetal brain development and increasing the risk of neurodevelopmental and behavioral disorders in childhood [10].

Health-related quality of life (HRQoL) provides a patient-centered framework to capture these multidimensional changes. The Short Form-36 Health Survey (SF-36) is widely used to assess HRQoL and has demonstrated sensitivity to changes in physical functioning, pain, and general health perceptions in obstetric populations [11]. Psychological distress, particularly anxiety, depression, and perceived stress, affects quality of life in pregnant women [11,12].

Maintaining healthy habits during pregnancy is essential for optimal pregnancy development and the prevention of associated pathologies [13]. These lifestyle behaviors also influence both physical and mental health dimensions. Research focuses on maternal nutrition, physical activity, and behavioral strategies, such as stress and anxiety management [14], smoking cessation [15], and quality sleep [16]. In addition, age, socioeconomic status, and education can directly influence maternal quality of life [17,18].

Despite strong evidence, routine mental health screening remains inconsistently implemented. Standard prenatal protocols often prioritize physical examinations, while emotional and psychosocial assessments are overlooked [19]. Integrated care models—combining obstetric services with psychological assessment and social support—have shown superior outcomes [20]. Collaborative frameworks involving obstetricians, midwives, psychologists, and social workers are particularly relevant in the post-COVID-19 era, where the burden on mental health has increased [20].

However, fewer studies have simultaneously examined both physical and mental HRQoL components during pregnancy and evaluated whether psychological distress is associated with these outcomes after accounting for key sociodemographic and clinical factors. To address this gap, the present study examines the association between psychological distress and both physical and mental HRQoL outcomes in pregnant women in Romania, including multivariable analyses adjusted for maternal age, trimester of pregnancy, education level, and medical comorbidities.

### 1.2. Objectives of Cross-Sectional Analysis

The following five research hypotheses are investigated in our analysis.

**H1.**  *Higher levels of psychological distress, measured by the Symptom Checklist-90-Revised (SCL-90-R) Global Severity Index (GSI), are associated with lower physical health scores measured by the Physical Component Summary (PCS) on the Short Form Health Survey-36 items (SF-36).*

**H2.**  *Higher levels of psychological distress (GSI) are associated with lower mental health scores measured by the Mental Component Summary (MCS) on the SF-36.*

**H3.**  *Reported alcohol measured by the Alcohol Use Disorders Identification Test—Consumption (AUDIT-C) or tobacco use measured by the Fagerström Test for Nicotine Dependence (FTND) is associated with lower HRQoL (PCS/MCS) on the SF-36 and high scores on the SCL-90-R GSI.*

**H4.**  *Pregnant women with medical comorbidities report higher psychological distress (SCL-90-R GSI) than those without medical comorbidities.*

**H5.**  *Pregnant women with medical comorbidities report lower HRQoL, both in physical (PCS) and mental (MCS) components of the SF-36, compared with those without medical comorbidities.*

We further examined whether these associations remained after adjustment for potential confounders in multivariable models.

## 2. Materials and Methods

### 2.1. Study Design and Setting

We conducted a cross-sectional survey at the First Clinical Department of Obstetrics and Gynecology (CDOG) of “Pius Brînzeu” Clinical County Hospital of Timișoara, Romania, between April 2023 and September 2025. The hospital is affiliated with “Victor Babeș” University of Medicine and Pharmacy of Timișoara and is a tertiary care center serving both urban and rural populations in western Romania, thus providing access to a diverse patient pool. All eligible patients were approached at the time of admission to minimize selection bias, and written informed consent was obtained prior to participation. After consent was provided, a member of the research team explained the instructions for completing each questionnaire and then provided participants with the paper-based questionnaires, which were completed in Romanian. Participants were given the opportunity to ask questions and request clarification if needed before and during questionnaire completion. The questionnaires took approximately 60 min to complete.

The study adhered to the STROBE guidelines for cross-sectional research and followed the ethical principles of the Declaration of Helsinki [21].

### 2.2. Participants

Eligible participants included pregnant women in their second or third trimester of gestation, aged 18 years or older, of Romanian nationality and residing in Romania, who provided written informed consent to participate. Exclusion criteria included multiple gestation, pre-existing severe psychiatric disorders and a history of drug addiction, and inability to provide informed consent.

**Figure 1:**
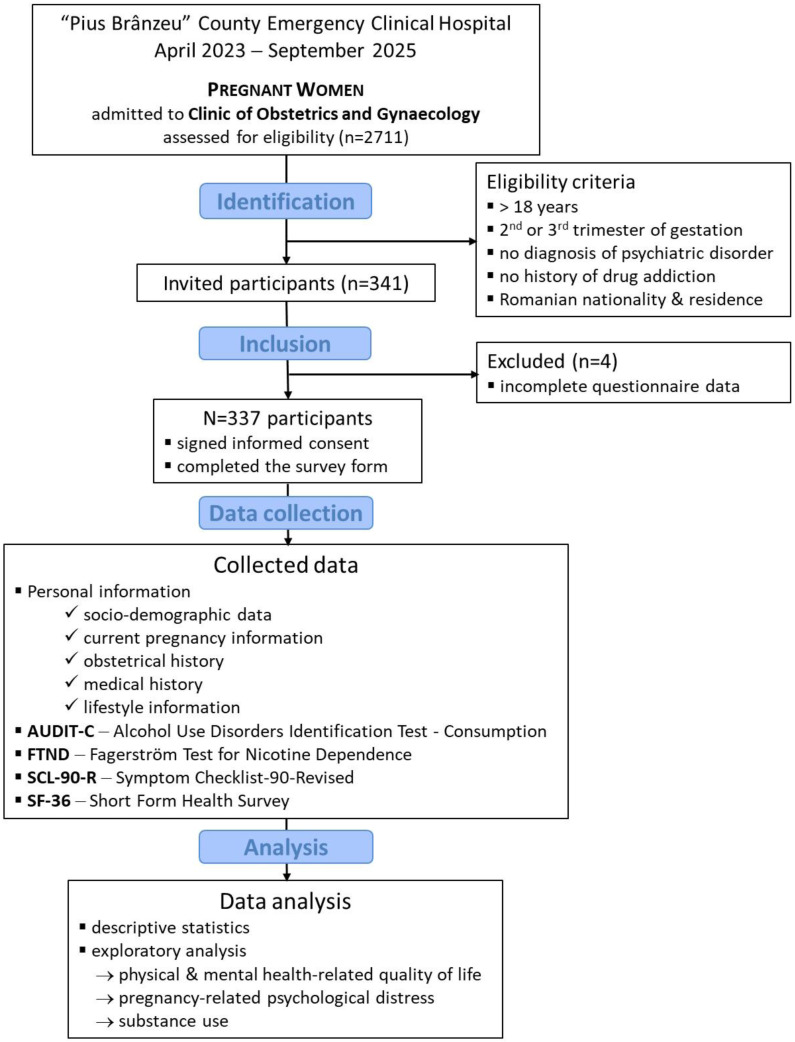
STROBE flow diagram of the selection process of participants.

Three hundred and forty-one women met the inclusion criteria. Four participants (1.2%) were excluded from the analysis due to incomplete questionnaire data for key study variables, resulting in a final analytic sample of 337 participants. The participant selection process is summarized in the STROBE flow diagram shown in Figure 1.

### 2.3. Variables

The primary outcome measures were the PCS and MCS scores derived from the SF-36 questionnaire, and psychological distress assessed using the SCL-90-R GSI. Secondary variables included demographic characteristics such as age, trimester of pregnancy, educational level, socio-economic status, medical comorbidities, and lifestyle factors (tobacco and alcohol use).

### 2.4. Measurement

Data collection relied on four internationally used questionnaires, complemented by a Personal Information Form (PIF) specifically developed for this study. We utilized instruments to which access was granted by the Department of Neurosciences, the Centre for Neuropsychology and Behavioural Medicine, the Centre for Social Diagnosis, the Psychiatry Clinic, and the CDOG in Timișoara.

Questionnaires were administered in Romanian using versions sourced from Vrasti [22] and guidance from the National Institute of Public Health [23]. Administration and scoring were standardized across participants, and data were checked for completeness and consistency. Internal consistency was evaluated in the present sample using Cronbach’s alpha, and findings involving measures with low reliability (AUDIT-C) were interpreted cautiously.

#### 2.4.1. PIF

The research team developed the PIF to collect the following information: sociodemographic characteristics (age, education level, residence, working conditions, and socio-economic conditions), obstetric history (current pregnancy, parity, miscarriages, and gestational age), and medical history (hypertension, diabetes mellitus, thyroid disorders, and other medical comorbidities). Medical comorbidities were initially self-reported by participants and subsequently verified using available medical records. Information on disease severity or specific clinical subtypes was not systematically collected. Socio-economic status was assessed as a self-reported four-level ordinal variable (low, lower-middle, upper-middle, or high) without predefined income thresholds.

#### 2.4.2. AUDIT-C

The AUDIT-C is a brief, 3-item screening tool derived from the longer AUDIT instrument, developed by the World Health Organization [24]. It assesses patterns of alcohol consumption, including drinking frequency, typical quantity, and episodes of heavy drinking. Each item is rated from 0 to 4, resulting in a total score between 0 and 12. For women, a cut-off score of ≥3 indicates hazardous drinking or possible alcohol use disorder. In our study, this scale presented a Cronbach’s α of 0.36.

#### 2.4.3. FTND

The FTND is a standardized 6-item questionnaire that evaluates physical dependence on nicotine [25]. The items evaluate key smoking behaviors, including time to first cigarette after waking, daily cigarette consumption, and difficulty refraining from smoking in restricted areas. Total scores range from 0 to 10, with higher scores indicating greater nicotine dependence. Scores are interpreted as follows: 0–2 = very low, 3–4 = low, 5 = medium, 6–7 = high, and 8–10 = very high. In our study, this scale presented a Cronbach’s α of 0.88.

#### 2.4.4. SCL-90-R

The SCL-90-R is a comprehensive self-report measure of psychological symptoms [26]. Participants rate 90 items on a 0–4 scale (from “not at all” to “extremely”) based on how much each symptom bothered them over the previous seven days. It measures nine primary symptom dimensions (and psychoticism) and three overall indicators of distress (GSI, PSDI, and PST). For the purposes of this study, we used the GSI as the primary summary measure; it demonstrated excellent internal consistency in our sample (Cronbach’s α = 0.97).

#### 2.4.5. SF-36

The SF-36 is a self-administered 36-item questionnaire that measures HRQoL [27,28]. The items are grouped into eight domains, which can be summarized into two composite indices: the Physical Component Summary (PCS) and the Mental Component Summary (MCS). The SF-36 has demonstrated excellent reliability and construct validity across multiple cultures and languages, including in obstetric cohorts, and is particularly sensitive in detecting HRQoL impairments during pregnancy. Cronbach’s alpha coefficient was not calculated for the PCS and MCS of the SF-36, as these are summary indices derived from the eight SF-36 domain scores using standardized (norm-based) scores and factor score coefficients (weights), rather than item-level scales that represent a single homogeneous construct [27,29].

### 2.5. Sample Size Justification

The sample size was estimated a priori using G*Power version 3.1 [30]. Based on previous studies reporting small to moderate effect sizes (ρ ≈ 0.20–0.25) for correlations between psychological distress and HRQoL in pregnant populations, a minimum of 194 participants were required to achieve 80% power at a 5% significance level. Our final sample of 337 participants provided adequate power (>95%) to detect small effect sizes in all planned analyses.

### 2.6. Bias and Missing Data Management

To minimize selection bias, recruitment followed a consecutive sampling approach throughout the study period. Data completeness was checked at the time of collection by trained research staff. Participants with missing data for the primary outcomes (SF-36 PCS/MCS or SCL-90-R GSI scores) were excluded (*n* = 4).

### 2.7. Statistical Analyses

Normality was assessed using the Shapiro–Wilk test; as most variables were nonparametrically distributed, nonparametric tests were applied to test hypotheses. Spearman correlations examined associations between continuous variables (e.g., GSI and SF-36 PCS/MCS scores). At the same time, Mann–Whitney U tests compared median psychological distress scores across study groups (e.g., with medical comorbidities vs. without medical comorbidities). All tests were two-tailed, with *p* = 0.05. Analyses were performed using IBM SPSS Statistics version v31.

In addition, multivariable adjusted analyses were conducted to account for potential confounding. Generalized linear models with robust standard errors were fitted separately for PCS and MCS outcomes. The main predictor was GSI, and models were adjusted for maternal age (years), trimester of pregnancy, education level, and the presence of medical comorbidities. Categorical covariates were entered as factors with appropriate reference categories. Model results are reported as unstandardized coefficients (B) with 95% confidence intervals (CI) and *p*-values.

Age was presented as <30/≥30 years in descriptive tables for readability; however, age was treated as a continuous variable in all statistical analyses.

## 3. Results

### 3.1. Participants and Descriptive Data

The study sample included 337 pregnant women, most of whom were in the third trimester. Participants classified as having medical comorbidities had diagnoses such as bronchial asthma, chronic bronchitis, chronic or gestational hypertension, type 2 or gestational diabetes mellitus, hyperthyroidism or hypothyroidism, tachycardia, anemia, peripheral varicose veins, or gastroduodenal ulcer.

Descriptive characteristics of the participants, including sociodemographic variables, obstetric data, presence of medical comorbidities, alcohol use, and tobacco dependence, are summarized in Table 1.

A summary of the comparison of socio-demographic and lifestyle characteristics between participants without associated pathologies (*n* = 155) and those with associated pathologies (*n* = 182) is presented in Table 2. Continuous variables are reported as mean ± standard deviation and were analyzed using the Mann–Whitney U test. For categorical variables, data are presented as *n* (%) within each group and were compared using the Pearson Chi-square test or Fisher’s exact test, where applicable. Participants with associated pathologies were significantly older than those without pathologies (40.90 ± 15.27 vs. 33.06 ± 8.63 years; *p* < 0.00). Consistent with this finding, the proportion of participants aged ≤30 years was lower in the group with medical comorbidities (28.0% vs. 40.0%; *p* = 0.02). No statistically significant differences were observed between groups in terms of type of residence, level of education, working conditions, socio-economic conditions, smoking, or alcohol consumption during pregnancy. The trimester of pregnancy showed a borderline difference between groups (*p* = 0.05).

### 3.2. Outcome Data and Main Results

Spearman’s rank-order correlations showed significant negative associations between psychological distress and HRQoL. Higher SCL-90-R GSI scores were associated with lower PCS scores, R = −0.16, *p* < 0.01, and lower MCS scores, R = −0.26, *p* < 0.01 (Table 3). No significant correlations were observed between alcohol or tobacco use and either PCS, MCS, or psychological distress scores.

The Mann–Whitney U test was used to test hypotheses H4 and H5, given the non-normal distribution of the variables. For H4, the analysis revealed that women with medical comorbidities (Mean Rank= 180.61) reported significantly higher psychological distress compared to those without medical comorbidities (Mean Rank= 155.36), U = 11,991.5, Z = −2.37, *p* = 0.02, r = 0.13. For H5, pregnant women with medical comorbidities reported significantly worse physical HRQoL scores on the SF-36 physical component compared with women without medical comorbidities (U = 11,639, Z = −2.77, *p* < 0.01, r = 0.15). In contrast, no significant differences were observed for the mental component of HRQoL (U = 13,734, Z = −0.42, *p* = 0.67) (Table 4 and Table 5).

Multivariable generalized linear models with robust standard errors were fitted for PCS and MCS component scores, adjusting for maternal age, trimester of pregnancy, education level, and the presence of pathologies (Table 6). Higher GSI was independently associated with lower PCS (B = −0.03, 95% CI [−0.06, −0.01], *p* = 0.003) and lower MCS (B = −0.03, 95% CI [−0.05, −0.01], *p* < 0.01). The absence of pathologies was associated with higher PCS compared with the presence of pathologies (B = 2.93, 95% CI [0.84, 5.02], *p* = 0.006), whereas pathologies were not significantly associated with MCS (B = −0.06, 95% CI [−1.30, 1.18], *p* = 0.92). Maternal age was positively associated with MCS (B = 0.10, 95% CI [0.06, 0.15], *p* < 0.01), but not with PCS (B = 0.06, 95% CI [−0.03, 0.14], *p* = 0.18). Trimester and education were not significantly related to PCS or MCS after adjustment (all *p* > 0.05).

## 4. Discussion

The results of this cross-sectional analysis of 337 pregnant women showed significant negative associations between psychological distress (measured using the Global Severity Index SCL-90-R) and HRQoL domains related to physical and mental health (assessed using the SF-36 instrument). Specifically, higher scores for psychological distress were related to lower scores on the PCS and MCS. Although the observed correlations (R = −0.16 and R = −0.26) were statistically significant, their magnitudes fall within the range conventionally considered “small” in the behavioral and health sciences, where correlation coefficients around 0.10 are regarded as small, around 0.30 as medium, and values above 0.50 as large [31]. This suggests that while psychological distress is associated with HRQoL scores, the practical or clinical impact of these relationships may be modest.

In addition, the presence of medical comorbidities was independently correlated with lower PCS scores, while older maternal age was positively correlated with MCS outcomes. In the adjusted models, the presence of medical comorbidities was associated with lower PCS, while older maternal age was positively associated with MCS outcomes. Notably, no significant associations were found between alcohol or tobacco use and HRQoL scores or psychological distress. It should be noted that the AUDIT-C scale showed low internal consistency in our sample (Cronbach’s α = 0.36), which may partly explain the lack of significant associations between alcohol use and HRQoL or psychological distress. This highlights the importance of addressing mental health in pregnant women to improve their overall well-being.

The SF-36 questionnaire captures several dimensions affected by mental health, including vitality, social functioning, and emotional roles. Longitudinal studies show a decline in scores across trimesters, particularly in individuals experiencing psychological distress [32,33]. Repeated-measures research using SF-36 has similarly shown that quality-of-life scores change across pregnancy, with a general decline as gestation advances [34].

The association between psychological distress and mental HRQoL is consistent with previous research showing that anxiety, depressive symptoms, and stress influence emotional well-being, vitality, and role functioning during pregnancy [35,36,37]. Psychological distress has also been linked to increased perception of somatic symptoms, fatigue, and reduced physical functioning, which may explain the observed association with physical HRQoL [32]. Importantly, in our multivariable models, the association between global psychological distress and both PCS and MCS remained statistically significant after adjustment for maternal age, trimester, education, and medical comorbidities. This suggests that psychological distress contributes to HRQoL beyond these key demographic and clinical factors.

The finding that women with medical comorbidities experienced greater psychological distress and poorer physical HRQOL is consistent with previous studies that have reported that chronic or pregnancy-related conditions, such as gestational diabetes, hypertensive disorders, or thyroid disease, increase symptom burden and pregnancy-related concerns [38]. These conditions can negatively impact physical functioning and overall health perceptions. Another study showed that among women with gestational diabetes who had abnormal SCL-90-R scores, they reported higher disease burden and lower well-being during pregnancy [39], further supporting the negative association between psychological distress and HRQoL. Consistent with this interpretation, comorbidities were independently associated with poorer PCS in the adjusted analysis, whereas their association with mental HRQoL (MCS) was not significant once overall psychological distress was accounted for. This pattern may indicate that mental HRQoL is more closely tied to the global symptom burden than to comorbidity status per se.

Unlike some previous studies that have identified alcohol and tobacco use as contributing factors to poorer maternal mental health and quality of life [40,41], no significant associations were observed in the present study. Possible explanations include low levels of reported substance use, underreporting due to social desirability bias, or insufficient variability to detect minor effects. Another possible explanation is that, in this clinical cohort, psychological stress and medical comorbidities exert a greater influence on perceived quality of life than lifestyle factors. These null associations were also unchanged in adjusted analyses, although measurement limitations (e.g., low AUDIT-C reliability) and low variability in reported use may have reduced power to detect small effects.

### 4.1. Clinical Implications

These findings support integrating brief, validated psychological screening into routine prenatal care, particularly for individuals who report elevated distress or present with medical comorbidities. Early identification of higher psychological symptom burden may facilitate timely referral to perinatal mental health services and targeted interventions (e.g., psychoeducation, stress-management strategies, and stepped-care support), with the potential to improve both mental and physical aspects of HRQoL.

In addition, the association between medical comorbidities and lower PCS underscores the importance of multidisciplinary management in complicated pregnancies. Clinically, patients with comorbidities may benefit from proactive counseling regarding symptom management, fatigue and sleep strategies, and functional support. Finally, given the modest effect sizes, interventions may be most impactful when targeted to higher-risk subgroups (e.g., those with high distress scores and concurrent comorbidities), where small average improvements can translate into meaningful gains in day-to-day functioning.

### 4.2. Strengths and Limitations

Key strengths of this study include the use of validated instruments for both psychological distress and HRQoL assessment, adherence to STROBE guidelines, and a relatively large, clinically relevant sample of 337 participants.

The cross-sectional design precludes causal inference and does not allow conclusions about the direction of the association between psychological distress and HRQoL. Although multivariable models adjusted for key covariates (age, trimester, education, and medical comorbidities), residual confounding by unmeasured factors (e.g., social support, prior mental health history, or major life stressors) cannot be excluded. Because participants were recruited from a single tertiary care center, the findings may not generalize to broader obstetric populations. Socio-economic status was self-reported without standardized income thresholds, which may have led to misclassification and limits comparability across studies. Finally, the AUDIT-C showed low internal consistency in our sample; therefore, null findings related to alcohol use should be interpreted cautiously.

## 5. Conclusions

The results of this study indicate that higher psychological distress is independently associated with lower HRQoL in both physical and mental domains. Women with medical comorbidities experienced higher levels of psychological distress and reduced physical HRQoL, while mental HRQoL did not vary significantly according to medical condition. In adjusted analyses, the presence of medical comorbidities was associated with poorer physical HRQoL, whereas mental HRQoL did not differ significantly by comorbidity status after accounting for overall psychological distress and sociodemographic factors. Maternal age was positively associated with MCS, but not with PCS, while trimester of pregnancy and education were not significantly related to either HRQoL component. These findings highlight the clinical value of incorporating routine mental health screening into prenatal care, particularly for women with medical comorbidities, and suggest that psychological distress may represent a key target for supportive interventions aimed at improving maternal well-being. Future longitudinal research is needed to clarify temporal relationships and to evaluate the effectiveness of psychosocial support during pregnancy.

## Figures and Tables

**Table 1:** Socio-demographic characteristics of the participants.

Baseline Characteristic	Full Sample
	*n* = 337
Age group	
≤30 years	113 (33.7%)
>30	224 (66.3%)
Residence type	
Urban	176 (52.2%)
Rural	161 (47.8%)
Educational level	
Primary school	11 (3.3%)
Middle school	25 (7.4%)
Vocational school	43 (12.8%)
High school	133 (39.5%)
Graduate school	85 (25.2%)
Postgraduate degree	40 (11.9%)
Working conditions	
Low-risk	243 (72.1%)
Medium-risk	68 (20.2%)
High-risk	26 (7.7%)
Socio-economic conditions	
Very good	69 (20.5%)
Good	197 (58.5%)
Satisfying	70 (20.8%)
Poor	1 (0.2%)
Trimester of pregnancy	
II	37 (11.0%)
III	300 (89.0%)
Associated pathologies	
No	155 (46.0%)
Yes	182 (54.0%)
Lifestyle	
Smoking during pregnancy	
No	256 (76.0%)
Yes	81 (24.0%)
Alcohol consumption	
No	248 (73.6%)
Yes	89 (26.4%)

**Table 2:** Socio-demographic of participants from the two groups.

Characteristic	Category	Medical Comorbidities: No (*n* = 155)	Medical Comorbidities: Yes (*n* = 182)	*p*-Value
Socio-demographic characteristics				
Age (years)	Mean ± SD	33.06 ± 8.63	40.90 ± 15.27	0.01 *
Age group	≤30 years	62 (40.0%)	51 (28.0%)	0.02 *
	>30 years	93 (60.0%)	131 (72.0%)	
Residence type	Urban	84 (54.2%)	92 (50.5%)	0.51
	Rural	71 (45.8%)	90 (49.5%)	
Educational level	Primary school	4 (2.6%)	7 (3.8%)	0.16
	Middle school	7 (4.5%)	18 (9.9%)	
	Vocational school	18 (11.6%)	25 (13.7%)	
	High school	58 (37.4%)	75 (41.2%)	
	Graduate school	46 (29.7%)	39 (21.4%)	
	Postgraduate degree	22 (14.2%)	18 (9.9%)	
Working conditions	Low-risk	117 (75.5%)	126 (69.2%)	0.43
	Medium-risk	27 (17.4%)	41 (22.5%)	
	High-risk	11 (7.1%)	15 (8.2%)	
Socio-economic conditions	Very good	34 (21.9%)	35 (19.2%)	0.40
	Good	94 (60.6%)	103 (56.6%)	
	Satisfying	27 (17.4%)	43 (23.6%)	
	Poor	0 (0.0%)	1 (0.5%)	
Trimester of pregnancy	II	23 (14.8%)	14 (7.7%)	0.05
	III	132 (85.2%)	168 (92.3%)	
Lifestyle				
Smoking during pregnancy	No	114 (73.5%)	142 (78.0%)	0.37
	Yes	41 (26.5%)	40 (22.0%)	
Alcohol consumption	No	114 (73.5%)	134 (73.6%)	1.00
	Yes	41 (26.5%)	48 (26.4%)	

Notes: Categorical variables are presented as *n* (%). Continuous variables are presented as mean ± SD; *p*-values for age were computed using Mann–Whitney U. For categorical variables, Fisher’s exact test or Chi-square test were applied for 2 × 2 tables. Percentages are within-group. * *p* < 0.5.

**Table 3:** Spearman correlations.

Variable	N	1	2	3	4	5
1. SF-36 PCS	337	-	0.28 **	−0.16 **	−0.04	0.12
2. SF-36 MCS	337	0.28 **	-	−0.26 **	0.03	−0.05
3. SCL-90-R GSI	337	−0.16 **	−0.26 **	-	0.02	0.07
4. AUDIT-C	337	−0.04	0.03	0.02	-	0.06
5. FTND	337	0.12	−0.05	0.07	0.06	-

Note: Correlations are Spearman’s *r_s_* coefficients. ** *p* < 0.01; MCS = the Mental Component Summary of Short Form Health Survey-36 items (SF-36); PCS = the Physical Component Summary of SF-36; SCL-90-R GSI = Global Severity Index of Symptom Checklist-90-Revised (SCL-90-R); AUDIT-C = Alcohol Use Disorders Identification Test-Consumption; FTND = Fagerström Test for Nicotine Dependence.

**Table 4:** Comparisons between thetwo groups.

Variable	U	Z	*p*	R (Effect Size)
SCL-90-R GSI	11,991.50	−2.37	0.02 *	0.13
SF-36 PCS	11,639	−2.77	0.01 **	0.15
SF-36 MCS	13,734	−0.42	0.67	

Note: The Mann–Whitney U test was used to compare groups of participants with and without associated pathologies. The effect size *r* was calculated using the formula $r=\frac{\left|Z\right|}{\sqrt{N}}$. Confidence intervals represent a bias-corrected 95% CI based on Fisher’s z transformation. * *p* < 0.05, ** *p* < 0.01.

**Table 5:** Descriptive statistics for comparisons between the two groups.

Variable	Group	N	Mean Rank	Sum of Ranks
SCL-90-R GSI	Without medical comorbidities	155	155.36	24,081.50
	With medical comorbidities	182	180.61	32,871.50
SF-36 PCS	Without medical comorbidities	155	153.09	23,729
	With medical comorbidities	182	182.55	33,224
SF-36 MCS	Without medical comorbidities	155	166.61	25,824
	With medical comorbidities	182	171.04	31,129

Note: N = the number of participants in each group. Mean ranks indicate score trends in each group, and the Mann–Whitney U test evaluates the differences between them.

**Table 6:** Multivariable generalized linear models with robust standard errors for PCS and MCS component scores.

Predictor	PCS (B, 95% CI)	*p*	MCS (B, 95% CI)	*p*
GSI	−0.03 (−0.06, −0.01)	0.01 **	−0.03 (−0.05, −0.01)	0.01 **
Age (years)	0.06 (−0.03, 0.14)	0.18	0.10 (0.06, 0.15)	0.01 **
Medical comorbidities	2.93 (0.84, 5.02)	0.01 **	−0.06 (−1.30, 1.18)	0.92
Trimester				
2nd vs. 3rd.	0.55 (−4.03, 5.13)	0.81	−0.74 (−3.16, 1.69)	0.55
Education				
Primary vs. Master/PhD	−3.64 (−10.47, 3.19)	0.29	−1.20 (−7.56, 5.16)	0.71
Middle school vs. Master/PhD	1.42 (−3.01, 5.85)	0.52	0.37 (−2.31, 3.04)	0.7
Vocational vs. Master/PhD	−1.86 (−5.85, 2.13)	0.36	−1.79 (−4.45, 0.87)	0.18
High school vs. Master/PhD	−1.70 (−4.98, 1.58)	0.31	−1.78 (−3.81, 0.26)	0.08
University vs. Master/PhD	−1.24 (−4.58, 2.09)	0.46	0.40 (−1.61, 2.42)	0.69

Note: B = unstandardized coefficient. Generalized linear models (normal distribution, identity link) with robust (sandwich) standard errors. Reference categories: trimester = 3rd; education = Master/PhD; medical comorbidities = Yes. Models adjusted for age, trimester, education, medical comorbidities, and GSI. ** *p* < 0.01.

## Data Availability

The original contributions presented in this study are included in the article. Further inquiries can be directed to the corresponding author.
